# Impact of Annual Praziquantel Treatment on Urogenital Schistosomiasis in a Seasonal Transmission Focus in Central Senegal

**DOI:** 10.1371/journal.pntd.0004557

**Published:** 2016-03-25

**Authors:** Bruno Senghor, Omar Talla Diaw, Souleymane Doucoure, Mouhamadane Seye, Adiouma Diallo, Idrissa Talla, Cheikh T. Bâ, Cheikh Sokhna

**Affiliations:** 1 Institute Recherche pour le Développement, UMR 198 (URMITE), Campus International de Hann, IRD, Dakar, Sénégal; 2 Université Cheikh Anta Diop de Dakar, Département de Biologie Animale, laboratoire d’écologie et de Biologie évolutive, Dakar, Senegal; 3 Institut Sénégalais de Recherches Agricoles, ISRA, Dakar, Senegal; 4 Programme national de lutte contre les bilharzioses et les géo-helminthiases, Ministère de la Santé et de l'Action sociale (MSAS), Dakar, Sénégal; Centers for Disease Control and Prevention, UNITED STATES

## Abstract

In Sub-Saharan Africa, urogenital schistosomiasis remains a significant public health problem, causing 150.000 deaths/year with approximately 112 million cases diagnosed. The Niakhar district is a disease hotspot in central Senegal where transmission occurs seasonally with high prevalences. The aim of this study was to determine the effect of annual treatment over 3 years on the seasonal transmission dynamics of *S*. *haematobium* in 9 villages in the Niakhar district. Adults and children aged between 5 and 60 years were surveyed from 2011 to 2014. Urine samples were collected door-to-door and examined for *S*. *haematobium* eggs at baseline in June 2011, and all participants were treated in August 2011 with PZQ (40 mg/kg). After this initial examination, evaluations were conducted at 3 successive time points from September 2011 to March 2014, to measure the efficacy of the annual treatments and the rates of reinfection. Each year, during the transmission period, from July to November-December, malacological surveys were also carried out in the fresh water bodies of each village to evaluate the infestation of the snail intermediate hosts. At baseline, the overall prevalence of *S*. *haematobium* infection was 57.7%, and the proportion of heavy infection was 45.3%, but one month after the first treatment high cure rates (92.9%) were obtained. The overall infection prevalence and proportion of heavy infection intensities were drastically reduced to 4.2% and 2.3%, respectively. The level of the first reinfection in February-March 2012 was 9.5%. At follow-up time points, prevalence levels varied slightly between reinfection and treatment from 9.5% in June 2012 to 0.3% in March 2013, 11.2 in June 2013, and 10.1% April 2014. At the end of the study, overall prevalence was significantly reduced from 57.7% to 10.1%. The overall rate of infested Bulinid snails was reduced after repeated treatment from 0.8% in 2012 to 0.5% in 2013. Repeated annual treatments are suggested to have a considerable impact on the transmission dynamics of *S*. *haematobium* in Niakhar, due to the nature of the epidemiological system with seasonal transmission. Thus, to maintain this benefit and continue to reduce the morbidity of urogenital schistosomiasis, other approaches should be integrated into the strategy plans of the National program to achieve the goal of urogenital schistosomiasis elimination in seasonal foci in Senegal.

## Author Summary

Urogenital schistosomiasis represents a serious cause of morbidity and mortality in *S*. *haematobium* endemic countries. Treatment with praziquantel (PZQ) is effective at reducing or eliminating active infection, but does not prevent reinfection that remains a continuing problem in high-risk communities, especially in areas where contacts with infested water are inevitable. The efficacy of PZQ treatment is challenged by regular re-infection in high risk communities due to frequent contact with water infested with snails carrying the larvae of *S*. *haematobium*. A large number of countries have initiated schistosomiasis control and elimination programs based on repeated PZQ mass drug administration (MDA). However, little information is available on the impact of repeated treatment on *S*. *haematobium* transmission dynamics in areas where transmission occurs seasonally in temporary water bodies. This observational study assessed the effect of annual repeated cohort treatment with PZQ over 3 years on the seasonal transmission dynamics of *S*. *haematobium* in nine villages in Niakhar district, District. Repeated annual treatments have a considerable impact on the transmission dynamics of *S*. *haematobium* in Niakhar. The prevalence of *S*. *haematobium* was dramatically reduced from 57.7% in 2011 to 10.1% in 2014. The reinfection rate did not exceed 10% from 2012 to 2014. Also, the rate of infested Bulinid snails decreased from 0.8% in 2012 to 0.5% in 2013. These results suggest that it may be possible to implement urogenital schistosomiasis elimination strategies in the Niakhar district and other seasonal transmission areas in Senegal if praziquantel MDAs are carried out in combination with other measures such as health education, improvement of access to clean water and snail control.

## Introduction

Urogenital schistosomiasis is a parasitic disease caused by infection with *Schistosoma haematobium*, *a* blood fluke of the genus *Schistosoma* [1]. In Africa, urogenital schistosomiasis remains a significant public health problem and causes 150.000 deaths / year with approximately 112 million cases diagnosed [2]. The transmission cycle requires contamination of fresh-water water by viable eggs passed in the urine of infected people, specific freshwater snails of the genus *Bulinus* as intermediate hosts and human water contact [1]. *S*. *haematobium* transmission is perennial in areas where fresh-water bodies are permanent and occurs seasonally in areas where they are temporary. Praziquantel (PZQ) is currently the drug of choice for treatment against *S*. *haematobium* with different regimes of PZQ administration implemented according to the prevalence of the disease [3, 4]. For example annual mass drug administration (MDA) of PZQ over five to six years is recommended according to the WHO guidelines when the prevalence of disease is ≥ 50% in school aged children (SAC) [5]. Recent studies showed that, except in the regions of Dakar and Thiès, the prevalences varied from 10% in the central regions with seasonal transmission, to over 95% in the Senegal River Basin (SRB) where the transmission is perennial [6, 7]. Along the SRB, treatment with PZQ has proved very effective at reducing *S*. *haematobium* infections; however prevalences can remain high due to the high abundance of permanent water bodies and persistent water contact that maintains transmission of the disease throughout the year [7]. Therefore, in such settings, despite repeated MDAs, the control of schistosomiasis transmission is very problematic as reinfection occurs rapidly and prevalence can return to pre-treatment levels quickly [7–11]. This situation differs from that observed in seasonal transmission areas where a single dose of PZQ (40 mg/kg) leads to a significant reduction in prevalence and intensity of infection of *S*. *haematobium* in SAC [12]. Efforts to control human schistosomiasis are gathering momentum and new ambitious goals for control and elimination have been put forward [13, 14]. Monitoring and evaluation of the impact of control programmes in different transmission foci is crucial for successful control in different epidemiological settings. In the area of Niakhar, in central Senegal, due to the lack of continued treatment, the prevalence of urogenital schistosomiasis increased to 73.2% from 56.7% in SAC [15, 16] indicating that continued PZQ MDA is needed to control the disease in this area. The aim of this study was to evaluate the impact of repeated annual PZQ treatments for three consecutive years on the dynamics of *S*. *haematobium* infection in Niakhar, where seasonal transmission takes place in temporary rain ponds and/or backwaters. In addition the effect of annual repeated treatments on the infestation of the snail intermediate hosts at the transmission sites was assessed.

## Methods

### Ethics statement

The study was approved by the Senegalese National Ethics Committee (reference: SN11/57). Local authorities of the Niakhar district were informed about the purpose, procedures, and potential risks and benefits of the study. The objectives of the study were explained to the participants when they were invited to participate. Written informed consent was obtained from all adult participants. For participants below 18 years of age, their parents or legal guardians provided written informed consent on their behalf. Only those who had a written informed consent were included. All infected subjects present at each treatment were offered PZQ (40mg/kg) and anyone absent was referred to the local health centers.

### Study area

**Fig 1:**
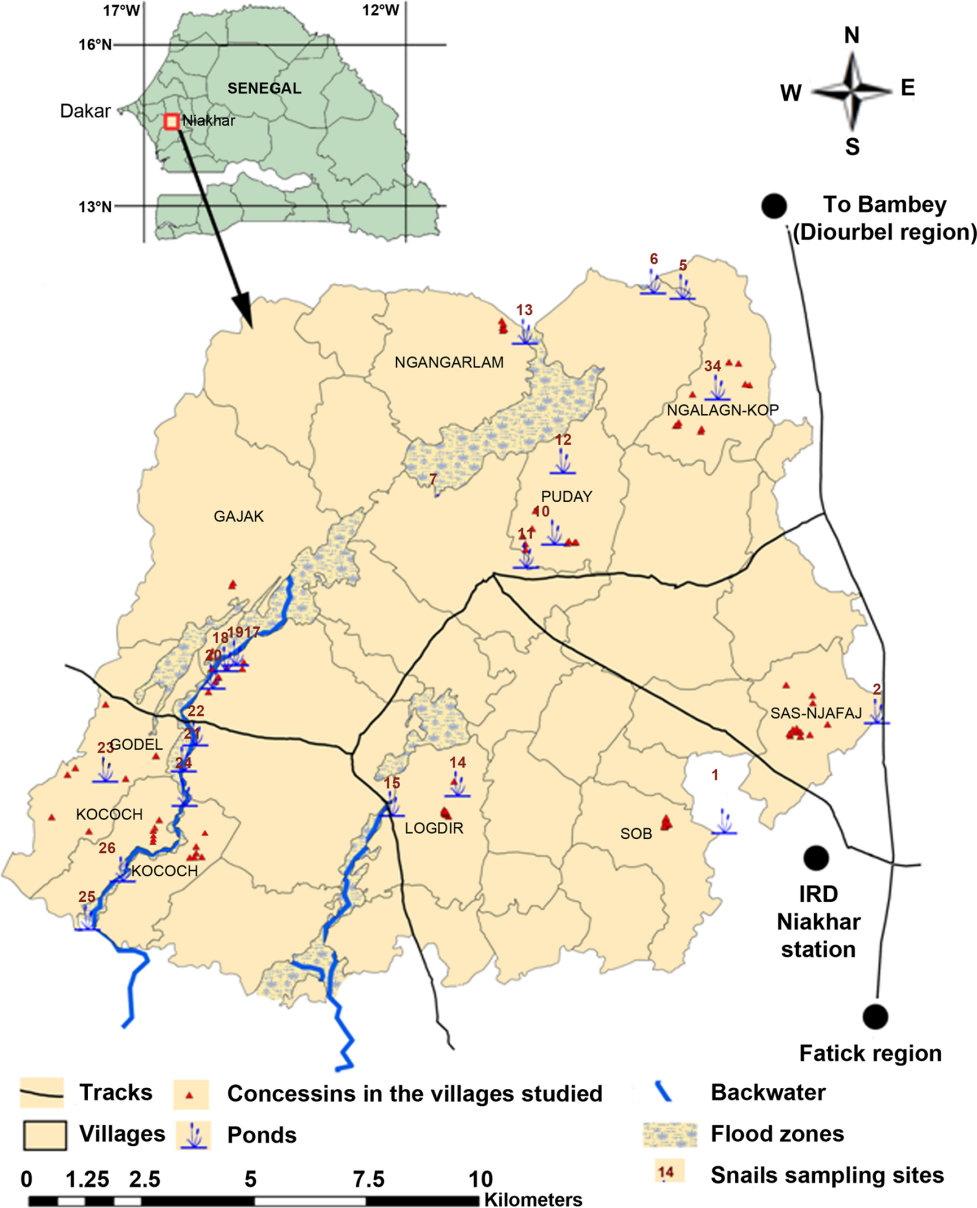
Map of the study area of Niakhar showing the villages studied and the snail sampling sites in the fresh-water ponds and backwaters.

The Niakhar district (14°30’ N, 16°30’ W) is located in the region of Fatick (west-central Senegal) and consists of 30 villages of ~44,000 inhabitants, covering a total area of 230 km^2^ [17] Here temporary ponds and backwaters are formed during the rainy season between July and November. These fresh water bodies are the only transmission sites for *S*. *haematobium* and are popular for domestic and recreational activities. From January until the end of June these water bodies are dry and so inhibit *S*. *haematobium* transmission. In December, few wet areas remain but they are not suitable for village activities [18]. The study population included SAC (5–15 years) and adults (15–60) years. They were enrolled from nine villages in the study area, Niakhar: Gajak (V1), Godel (V2), Kocokh (V3), Logdir (V4), Ngalagne kop (V5), Ngangarlam (V6), Puday (V7), Sass njafaj (V8) and Sob (V9) (Fig 1). The nine villages were enrolled based their *S*. *haematobium* prevalence, type of water bodies and access to safe tap water as previously reported [16]. Bathing, swimming, fetching water, laundry, washing pets and fishing are the main water contact activities in these areas. The villages of Gajak, Godel and Kocokh have no access to tap water and are crossed by a large backwater near the households. The populations of these three villages use the backwater in the rainy season and wells in the dry season. The backwater is salty in the villages of Kocokh and Godel but the population from Godel also uses one pond located inside the village. In the villages of Logdir, Ngalagne kop, Ngangarlam, Puday, Sass njafaj and Sob, all water-related activities except fishing are carried out in the ponds. The hydrological network is very dense at Ngalagne kop, Logdir and Puday where the ponds are very close to the households. At Sass njafaj and Sob, ponds are scarce and far from the households. At Ngangarlam, ponds are also scarce but close to the households (Fig 1). Among the six villages using ponds, only Ngangarlam has no access to safe tap water. During the dry season, people in the villages without pipe borne water use water from the well or migrate to other villages were clean water is available. Before this study, the highest prevalence of *S*. *haematobium* (> 90%) was recorded in 2009 in the village of Kocokh and the lowest (< 25%) in the village of Sob [18]. No MDA had been implemented in these areas before the beginning of this study in 2011 and alongside this study MDA was carried out by the National Program in April 2012 and 2013. A more detailed description of the Niakhar district has been published elsewhere [16, 17].

### Study sample selection

The minimum sample size for this study was calculated based on the normal distribution N = ε^2^pq/i^2^. With N being the size of the sample; ε the normal Z score corresponding to the risk of error α = 5%; p the mean prevalence of *S*. *haematobium* (57.6%) in the study area [16]; q = (1—p); and i the precision, fixed at 5%. N = (1.96)^2^ (0.5) (0.42) / (0.05) ^2^ = 325. Taking into account losses due to absences, desertion, migration and others factors, estimated at 5%, the sample size should be: N = 325 + 325 x 5 / 100 = 341. Each village was considered as a cluster. Considering the cluster effect, such that children living in the same household could incur the same risks for *S*. *haematobium* infection, the sample size was multiplied by 2 in order to limit the cluster effect. N = 341 x 2 = 682. The sample size in each village was estimated according to the weight of its population in relation to other villages.

### Study design

A quantitative assessment of a cohort of the population was conducted in Niakhar district in central Senegal from June 2011 to May 2014. This study consisted of two major longitudinal surveys of *S*. *haematobium* infection: a parasitological survey of human populations in the village during the dry season and a malacological survey of the snail population in the transmission sites during the rainy season. The inclusion criteria were: i) to reside in the study villages during the rainy season ii) to be aged 5 to 60 years and iii) to consent to participate in the study.

**Fig 2:**
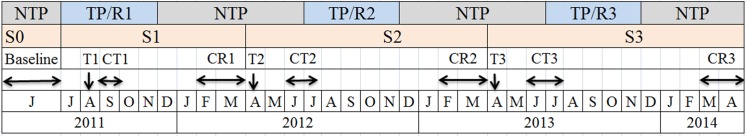
Calendar of parasitological survey (S) and treatment (T). The period from July to November corresponds to the transmission period (TP) and reinfection (R) (presence of water in the ponds and backwaters). The period from December to June corresponds to the non-transmission period (NTP) (dry period of water bodies). S0: Baseline survey (June 2011). S1: First treatment (T1); monitoring the efficacy of T1 (CT1); Control of the first reinfection (CR1). S2: Second treatment (T2); monitoring the efficacy of T2 (CT2); Control of the second reinfection (CR2). S3: T3: third treatment; monitoring the efficacy of T3 (CT3); Control of the third reinfection (CR3)

The parasitological survey was conducted door-to-door in four successive phases, S0, S1, S2 and S3 (Fig 2). S0 corresponds to the baseline parasitological surveys and was conducted in June 2011 during the period of non transmission of *S*. *haematobium*. The following phases S1, S2 and S3 were done between August 2011-March 2012, April 2012-March 2013 and April 2013-April 2014, respectively. The same individuals are tested and followed from one year to the next. Each phase included a single treatment (T) with a dose of 40 mg/kg of PZQ using the dose pole. The T1 treatment was conducted in August 2011 and involved the whole village population of the study. Treatments T2 (April 2012) and T3 (April 2013) only involved infected individuals.

PZQ efficacy was measured by determining the CR and the change in the proportion of heavy intensity of infection (≥ 50 eggs/10 ml of urine) after treatment. The CR is the percentage of participants becoming egg negative after PZQ treatment [19]. The evaluation of reinfection (R) involved *S*. *haematobium*-negative individuals before and after each treatment that had become infected after the transmission period. The reinfection was monitored during the dry season after the main transmission period, giving time for all juvenile worms to become adults and produce eggs. A participant was considered infected or reinfected by *S*. *haematobium* if at least one egg was found in the urine sample during microscopic examination. For the S1 phase, the CR1 and R1 rates were evaluated in September 2011 and between February and March 2012, respectively. The monitoring of the CR2 and R2 associated with S2 were done in June-July 2012 and February-March 2013, respectively. The CR3 and the R3 associated with S3 were evaluated in June-July 2013 and March-April 2014, respectively. The prevalence of *S*. *haematobium* was determined considering the whole study population after each PZQ efficacy monitoring and reinfection control. P1, P2, P3, P4, P5, and P6 are the successive overall prevalence in the population after CR1, R1, CR2, R2, CR3 and R3 respectively. The design of the study is detailed in Fig 2.

### Urine sample collection and microscopic detection of *S*. *haematobium* eggs

Urine samples were collected door-to-door from all subjects present in the household to evaluate the baseline prevalence and the CRs and R rates after the 3 treatments T1, T2 and T3. Plastic screw-vials were given to each participant to provide one urine sample between 10:00 am and 2:00 pm. The samples were transferred to the Niakhar laboratory and analyzed the same day. Microscopic assessment of each urine sample for identification of *S*. *haematobium* eggs was performed using the standard filtration method [20]. Each urine sample was shaken to ensure dispersal of eggs and ten milliliters (10 ml) were filtered through a Millipore SX0001300 Swinnex syringe filter. A drop of Lugol’s iodine was added to the filter before examination under a microscope slide for the presence of *S*. *haematobium* eggs. The presence and number of *S*. *haematobium* eggs per filter were recorded. Egg count was recorded as number of eggs per 10 ml of urine. The World Health Organization criterion was used to classify the intensity of the infection: light (1–49 eggs/10 ml of urine) or heavy (≥ 50 eggs/10 ml of urine) [5].

### Snail sampling and cercarial shedding test

Each year, during the transmission period, from July to November-December, parallel malacological surveys were carried out in each village to evaluate the infestation of the snail intermediate hosts. The snails were collected at transmission sites from fresh water bodies around each village and placed in plastic pots containing a few aquatic plants and water from the site to prevent them from dying during the transport to the laboratory at Niakhar for morphological identification. Snails were morphologically identified using a key for snail identification [21]. Snails were checked for infection using the cercarial shedding test. Individual snails were placed in glass tubes in 10 mls of filter water and incubated in indirect sunlight or electric light for 30 to 40 minutes to stimulate cercarial shedding. The glass tubes were then checked under a dissecting microscope and the presence of schistosome cercariae was recorded according to the morphological criteria developed by Fransden & Christensen [22].

### Data analysis

Data from each village were recorded using Epi-Info software, version 3.5.1 (August 13, 2008) and analyzed using STATA 11.1. At each time point, only individuals who attended the follow-up surveys were included in the longitudinal analysis. The relationships between human infection prevalence, intensity of infection and other variables, such as villages, sex and age of subjects, were tested at baseline. Prevalence, re-infection levels and intensity of *S*. *haematobium* infection before and after each treatment were compared using the 2×2 chi-squared test or the Fisher exact test [23]. For the comparison of the CR and R rates, Pearson chi-squared tests of proportions were carried out. In all cases, a *p*-value < 0.05 was taken to indicate statistical significance.

## Results

### Characteristics of the study population

A total of 623 subjects provided a urine sample at baseline. Both female (51.4%) and male (48.6%) participants provided samples. The largest proportion of participants was recruited from the village of Gajak (22.2%) and the smallest proportion in Sob (7.4%). Samples were analyzed from SAC (5–5 years) 67.6% and the adults (over 15 years) 32.4%. Table 1 summarizes the demographic characteristics of the participants. The reduction in the numbers of subjects at each time point was due to absence, non-cooperation or death.

**Table 1:** General characteristics of the study cohort.

Attributes		Number	Frequency (%)
Sex	Male	303	48.6%
	Female	320	51.4%
Age groups	5–15 years	421	67.6%
	Over 15 years	202	32.4%
Villages (Annotation)	Gajak (V1)	138	22.2%
	Godel (V2)	69	11.1%
	Kocokh (V3)	87	14.0%
	Logdir (V4)	69	11.1%
	Ngalagne kop (V5)	57	9.1%
	Ngangarlam (V6)	38	6.1%
	Puday (V7)	67	10.8%
	Sass ndiafaj (V8)	53	8.5%
	Sob (V9)	46	7.4%

### Baseline prevalence and intensity of *S*. *haematobium* infection at S0

Across all villages (623 participants surveyed) 359 (57.6%) were *S*. *haematobium* egg positive and prevalences ranged from 35.2% in V9 to 92.9% in V5 with heavy infection intensity varying from 92.9% in V5 to 22.2% in V9. A significant difference in the prevalence and intensity of infection was observed between villages (*p*< 0.001) (Table 2).

**Table 2:** Baseline *S*. *haematobium* infection, efficacy of three treatments of praziquantel and reinfection levels over three years in nine villages of the Niakhar study area.

Survey (S)	Variables	Total	V1	V2	V3	V4	V5	V6	V7	V8	V9	p-value
Baseline (P0)												
S0	No. Infected/examined	359/623	95/138	36/69	37/87	34/69	53/57	23/38	42/67	23/53	16/45	
	P0 in % (CI95)	57,6 (53.7–61.4)	68.8 (60.6–65.9)	52.2 (40.6–63.5)	42.5 (32.6–53.0)	49.3 (37.8–60.8)	92.9 (83.3–97.4)	60.5 (46.7–74.4)	62.7 (57.73.3)	43.4 (30.9–56.7)	35.2 (23.2–50.2)	0.003
	No. (%) ≥ 50 eggs/10 ml of urine	283 (45.4)	69 (50)	23 (33.3)	26 (29.9)	24 (34.9)	53 (92.9)	22 (57.9)	35 (52.2)	21 (39.6)	10 (22.2)	<0.001
First treatment T1 (August 2011)- CR1: follow-up five weeks in September 2011												
S1	No. infected and treated	351	92	36	36	34	51	23	40	23	16	
	No. cured (CR1 in %)	326 (92.9)	85 (92.4)	35 (97.2)	35 (97.2)	34 (100)	46 (90.2)	14 (60.7)	39 (97.5)	22 (95.6)	16 (100)	0.867
	P1 in % (CI95)	4.2 (2.9–6.1)	5.2 (2.5–10.3)	1.4 (0.2–7.7)	2.6 (0.6–7.9)	0 (0.0–5.2)	10.5 (4.9–21.1)	21.6 (11.4–37.2)	1.5 (0.3–7.9)	1.9 (0.3–9.9)	0 (0.0–7.8)	0.641
	No. (%) ≥ 50 eggs/10 ml of urine	11 (1.8)	2 (1.5%)	0	2 (2.6)	0	3 (5.3)	4 (10.8)	0	0	0	0.001
	Reinfection R1 (July to November 2011). R1: Follow-up in February to March 2012											
	No. infected/examined	61/617	15/135	2/69	0/87	3/69	11/56	11/37	15/66	1/53	3/45	
	P2 in % (CI95)	9.9 (7.7–12.5)	11.2 (6.8–17.5)	2.9 (0.7–9.9)	0 (0.0–4.2)	4.3 (1.5–12.0)	19.6 (11.3–31.8)	29.7 (17.5–45.9)	22.7 (14.3–34.2)	1.9 (0.3–9.9)	6.7 (2.3–17.9)	<0.001
	No. (%) ≥ 50 eggs/10 ml of urine	30 (4.9)	10 (7.4%)	1 (1.4)	0	2 (2.9)	2 (3.6)	6 (16.2)	6 (9.1)	1 (1.9)	2 (4.4)	<0.001
	No. reinfected/examined	40/592	9/128	1/68	0	3/69	3/29	3/29	14/65	0/52	3/45	
	R1 in % (CI95)	6.7 (5.0–9.1)	7 (3.7–12.8)	1.4 (0.2–7.8)	0	4.3 (1.5–12.0)	10.3 (3.6–26.4)	10.3 (3.6–28.4)	21.5 (13.3–32.9)	0	6.7 (2.3–17.9)	<0.001
Second treatment T2 (April 2012). CR2: Follow-up in June—July 2012 (3 to 4 months after treatment)												
S2	No. infected and treated	61	15	2	0	3	11	11	15	1	3	
	No. cured (CR2 in %)	58 (95.1)	15 (100)	2 (100)	0	3 (100)	10 (90.9)	10 (90.9)	14 (93.3)	1 (100)	3 (100)	0.091
	P3 in % (CI95)	0.4 (0.2–1.4)	0 (0.0–2.7))	0 (0.0–5.3)	0	0 (0.0–5.3)	1.8 (0.3–9.4)	1 (1.8)	1.5 (0.2–8.2)	0	0	0.210
	No. (%) ≥ 50 eggs/10 ml of urine	1 (0.2)	0 (0)	0 (0)	0	0	0	0	1 (1.5)	0	0	0.390
	Reinfection R2 (July to November 2012). R2: Follow-up in February to March 2013											
	No. Infected/examined	68/611	12/134	7/69	0/87	2/68	4/56	13/37	21/65	0/53	9/44	
	P4 in % (CI95)	11.1 (8.8–13.6)	8.9 (5.2–15)	10.1 (5.0–19.5)	0	2.9 (0.8–10.1)	7.1 (2.8–16.9)	35.1 (21.8–51.2)	32.3 (22.2–44.5)	0.0 (0.0–6.7)	20.4 (11.1–34.5)	<0.001
	No. (%) ≥ 50 eggs/10 ml of urine	41 (6.7)	9 (6.7)	5 (7.2%)	0	1 (1.5)	2 (3.5)	10 (27.0)	11 (16.9)	0	3 (6.8)	0.005
	No. reinfected/examined	66/609	12/134	7/69	0	2/68	3/56	12/36	21/64	0/53	9/44	
	R2 in % (CI 95)	10.8 (8.6–13.6)	8.9 (5.2–15)	10.1 (5.0–19.5)	0	2.9 (0.8–10.1)	5.3 (1.8–14.6)	33.3 (20.9–43.6)	32.8 (22.6–40)	0 (0.0–8.4)	20.4 (11.1–34.5)	<0.001
Third treatment T3 (April 2012)- CR 3: Follow-up in June—July 2013 (3 to 4 months after treatment)												
S3	No. infected and treated	67	12	7	0	2	4	12	21	0	9	
	No. cured (CR3 in %)	62 (95.5)	11 (91.7%)	7 (100)	0	2 (100)	4 (100)	10 (83.3)	20 (95.2)	0	8 (88.9)	<0.001
	P5 in % (CI95)	0.8 (0.3–1.9)	0.7 (0.1–4.1)	0 (0.0–5.3)	0	0 (0.0–5.3)	0 (0.0–6.4)	2 (5.4)	1 (1.5)	0	1 (2.3)	0.079
	No. (%) ≥ 50 eggs/10 ml of urine	3 (0.5)	1 (0.7)	0 (0)	0	0	0	2 (5.4)	0	0	0	0.008
	Reinfection R3 (July to November 2013). R3: Follow-up in March to April 2014											
	No. Infected/examined	61/611	20/134	1/69	0/86	10/68	2/56	9/37	7/53	7/53	2/44	
	No. infected (P6%)	9.9 (7.8–12.6)	14.9 (9.8–21.9)	1.4 (0.2–7.8)	0	14.7 (7.9–25.1)	3.6 (0.9–12.1)	24.3 (13.4–40.1)	13.2 (6.5–24.8)	13.2 (6.5–24.8)	4.5 (1.2–15.1)	<0.001
	No. (%) ≥ 50 eggs/10 ml of urine	41 (6.7)	14 (10.4)	1 (1.4)	0	6 (8.8)	0	6 (16.2)	2 (3.8)	5 (9.4)	1 (2.3)	<0.001
	No. reinfected/examined	61/606	20/133	1/69	0	10/68	2/56	9/35	10/63	7/53	2/43	
	R3 in % (CI 95)	10.1 (7.9–12.7)	15 (9.9–22.1)	1.4 (0.2–7.8)	0	14.7 (7.9–25.1)	3.6 (0.9–12.1)	25.7 (14.2–40.1)	15.8 (10.8–25.8)	13.2 (6.5–24.8)	4.6 (1.3–15.4)	<0.001

No: number; P0 to P6: Prevalences at each time points; R1, R2 and R3: reinfection rates; CR: cure rates; CI: confidence interval

Significant difference was observed in the prevalence and proportion of high intensity infections between males (69.3%, 63%) and females (46.1%, 28.7%) (*p* < 0.05) with prevalence and high intensity infections significantly decreasing with age (71.7%, 86.6% in 1–5 year olds and 28.2%, 7.6% in 5–60 year olds) (*p*< 0.001) (Table 3).

**Table 3:** Baseline *S*. *haematobium* infection, efficacy of three treatments of praziquantel and reinfection levels over three years according to age group and sex.

Survey (S)	Variables	Total	5–15 years	Over 15 years	*p*-value	Male	Female	p-value
Baseline (P0)								
S0	No. infected/examined	359/623	302/421	57/202		210/303	149/320	
	P0 in % (CI95)	57.6 (53.7–61.4)	71.7 (67.2–75.8)	28.2 (22.5–34.8)	<0.001	69.3 (64.5–74.3)	46.1 (41.1–52.0)	<0.001
	No. (%) ≥ 50 eggs/10 ml of urine	283 (45.4)	260 (61.7)	23 (11.4)	<0.001	191 (63)	92 (28.7)	<0.001
First treatment T1 (August 2011)- CR1: Follow-up five weeks in September 2011								
S1	No. infected and treated	351	296	55		203	148	
	No. cured (CR1 in %)	326 (92.9)	272 (91.9)	54 (98.2)	0.084	181 (89.2)	145 (97.9)	0.359
	P1 in % (CI95)	4.2 (2.9–6.1)	5.9 (4.1–8.6)	0.5 (0.1–2.8)	0.002	9.6 (6.7–13.4)	1.3 (0.5–3.2)	<0.001
	No. (%) ≥ 50 eggs/10 ml of urine	11 (1.8)	11 (2.6)	0 (0)	0.021	11 (3.6)	0 (0)	0.001
	Reinfection R1 (July to November 2011). R1: Follow-up in February to March 2012							
	No. infected/examined	61/617	59/418	2/199		48/298	13/319	
	P2 in % (CI95)	9.9 (7.7–12.5)	14.1 (11.1–17.8)	1 (0.1–2.8)	<0.001	16.1 (12.7–20.7)	4.1 (2;3–6.8)	<0.001
	No. (%) ≥ 50 eggs/10 ml of urine	30 (4.9)	30 (7.2)	0 (0)	<0.001	24 (8.1)	6 (1.9)	<0.001
	No. reinfected /examined	40/592	38/393	2/199		30/181	10/145	
	R1 in % (CI95)	6.7 (5.0–9.1)	9.6 (7.1–12.9)	1 (0.1–2.8)	<0.001	16.6 (11.8–22.6)	6.9 (3.7–12.2)	<0.001
Second treatment T2 (April 2012). CR2: Follow-up in June—July 2012 (3 to 4 months after treatment)								
S2	No. infected and treated	61	59	2		48	13	
	No. cured (CR2 in %)	58 (95.1)	56 (96.6)	2 (100)	0.259	45 (93.8)	13 (100)	0.480
	P3 in % (CI95)	0.4 (0.2–1.4)	0.7 (0.2–2.8)	0 (0.0–1.9)	0.487	1 (0.3–2.9)	0 (0.0–1.9)	0.216
	No. (%) ≥ 50 eggs/10 ml of urine	1 (0.2)	1 (0.2)	0 (0)	0.490	1 (0.3)	0 (0)	0.330
	Reinfection R2 (July to November 2012). R2: Follow-up in February to March 2013							
	No. infected/examined	68/611	65/414	3/197		46/298	22/313	
	P4 in % (CI95)	11.1 (8.8–13.6)	15.7 (12.5–19	1.5 (0.5–4.4)	<0.001	15.4 (11.8–20)	7 (4.7–10.4)	0.003
	No. (%) ≥ 50 eggs/10 ml of urine	41 (6.7)	40 (9.7)	1 (0.5)	<0.001	27 (9.1)	14 (4.5)	0.036
	No. reinfected /examined	66/609	63/412	3/197		44/296	22/313	
	R2 in % (CI 95)	10.8 (8.6–13.6)	15.3 (12.1–19.1)	1.5 (0.5–4.4)	<0.001	14.9 (11.3–19.4)	7 (4.7–10.4)	0.002
Third treatment T3 (April 2012)- CR 3: Follow-up in June—July 2013 (3 to 4 months after treatment)								
S3	No. infected and treated	67	64	3		45	22	
	No. cured (CR3 in %)	62 (95.5)	59 (92.2)	3 (100)	0.561	42 (93.3)	20 (90.1)	0.728
	P5 in % (CI95)	0.8 (0.3–1.9)	1.2 (0.5–2.8)	0 (0.0–1.9)	0.187	1 (0.3–2.9)	0.6 (0.2–2.3)	0.880
	No. (%) ≥ 50 eggs/10 ml of urine	3 (0.5)	3 (0.7)	0 (0)	0.121	3 (1)	1 (0.3)	0.612
	Reinfection R3 (July to November 2013). R3: Follow-up in March to April 2014							
	No. Infected/examined	61/611	60/414	1/197		40/298	21/313	
	P6 in % (CI95)	9.9 (7.8–12.6)	14.5 (11.4–18.3)	0.5 (0.1–2.8)	<0.001	13.4 (10–17.8)	6.7 (4.4–10)	0.014
	No. (%) ≥ 50 eggs/10 ml of urine	41 (6.7)	40 (9.7)	1 (0.5)	<0.001	28 (9.4)	13 (4.1)	0.016
	No. reinfected/examined	61/606	60/409	1/197		40/295	21/311	
	R3 in % (CI 95)	10.1 (7.9–12.7)	14.7 (11.6–18.4)	0.5 (0.1–2.8)	<0.001	13.6 (10.1–17.9)	6.7 (4.5–10.1)	0.006

### Impact of PZQ treatment on annual *S*. *haematobium* transmission dynamic

**S1**. Among the 359 infected individuals at T0, 351 were treated at T1 and monitored for PZQ efficacy. Eight subjects were not treated due to their absence. Overall, a high cure rate (CR1) of 92.9% (326/351) was observed with an average prevalence of 4.2% across all villages. At the village level, a significant decrease of the prevalence was observed in all villages after the 1st treatment T1 (*p*< 0.05) ranging from a high of 21.5% in V6 to 0% in V4 and V9 (Table 2). CR1's were between 90% and 100% except for V6 where the CR1 was 60.1%. No significant difference was observed between villages (*p*> 0.05) (Table 2).

Heavy infections were eliminated in all villages except V1 (1.5%) and V6 (10.8%).

There was no significant difference in CR1 according to age and sex (*p*> 0.05) and CR1 was high in both SAC (91.9%) and adults (98.2%), females (97.9%) and males (89.2). Heavy infections were eliminated after T1 in adults and in female SAC. Reduction in prevalence and proportion of heavy infections after T1 was strongly correlated to age and sex of the infected individuals (*p*< 0.05) (Table 3).

The overall re-infection (R1) rate during the 2011 transmission season was 6.7%. No reinfection occurred in V3 and V8 during this period. In the other villages, R1 rates were significantly different (*p* < 0.05) in each village, ranging from 1.4% in V2 to 21.5% in V7. However the overall prevalence after R1 was significantly lower compared to baseline prevalence (*p* < 0.05) (Table 2). The rate of reinfection was significantly higher in SAC (9.6%) than in adults (1%) and also in males (16.6%) compared to females (6.9%) (*p* < 0.05) (Table 3).

**S2**. During S2, a total of 61subjects were found positive and received treatment at T2. Among these 61 individuals, 58 became egg negative (95.1%) while only 3 individuals remained infected; one each in V5, V6 and V7. Therefore, after T2 no reinfection was observed in 6 out of the 9 villages. No significant difference in CR2 was observed between villages and was comparable to CR1. Heavy infections were eliminated in all villages except V7, where only one male SAC excreted over 49 eggs/10 ml of urine. After the 2012 transmission season, the overall R2 rate was 10.8% and higher than R1 (6.7%) (*p* > 0.05). No reinfection was observed in V3 and V8 but ranged from 33.3% in V6 to 2.9% in V4 with a significant difference in R2 between villages (*p*< 0.05). Comparing the R2 and R1 rates, it would appear that the trend was not the same across villages. V1, V2, V6, V7 and V9 experienced an increase in reinfection rate from R1 to R2, whereas V4 and V5 experienced a decrease. The heavy infections increased in seven villages and varied between 1.5% at V4 to 27.0% at V6, but remained significantly lower than at baseline (*P*< 0.05) (Table 2).

The R2 rate was 15.7% and 1.5% in SAC and adults, respectively and significantly higher in males (14.9%) than in females (7%) subjects (*p*< 0.001). The difference between R2 and R1 was only significant in the SAC group (*p*< 0.05). There was no significant change in the proportion of heavy *S*. *haematobium* infections between R1 and R2 according to age or sex (Table 3).

**S3.** During S3, 67 infected individuals were treated (T3). The CR3 was high at 95.5% (62/67) and we did not detect *S*. *haematobium* reinfection occurred in V2, V4, V7 and V8. In villages V1, V6, V7 and V9, the prevalence was significantly reduced compared to R2 (*p* < 0.05) (Table 2). Heavy intensity infections were absent in all villages except V1 and V6, where 1 and 2 SAC respectively had more than 49 eggs/10 ml of urine. No significant difference in CR3 was observed between villages and in any village between CR1 and CR3 and between CR2 and CR3 (*p*> 0.05). There was no significant difference of CR according to age and sex (*p* > 0.05). The CR3 was 92.2% and 100% in SAC and adults, respectively and 93.3% and 90.1% in males and females respectively (Table 2).

At the end of 2013 the transmission season, except in V3, where no *S*. *haematobium* egg positive individuals were found, reinfection occurred in all other villages and ranged from 1.4% in V2 to 25.7% in 25.7 in V6. There was a significant difference in R3 between villages (*p* < 0.05) and a significant increase in reinfection between R1 and R3 in V1, V4 and V8 was observed. The same trend was also found between R2 and R3 in V4, V5, and V8 (*p* < 0.05). In V6 and V7, where R1 and R2 were higher than in other villages, there was a reduction of R3 compared to R1 and R2, though a statistically significant difference was only noted in V6 (*p* < 0.05). Overall, R3 was significantly higher than R1 (*p* < 0.05) but there was no significant difference compared to R2 (*p* > 0.05). A significant increase of prevalence after R3 was noted in V1, V4, V7 and V8 (*p* < 0.05). However, the overall prevalence remained significantly lower than at baseline in each village (*p* < 0.05) (Table 2).

The same trend of reinfection was noted between ages for R3, with SAC (14.7%) having a higher R3 rate than adults (0.5%) and also between males (13.6%) and females (6.7) (*p* < 0.05). There were no differences between R3 and R2, but R3 was significantly higher than R1 for SAC (*p* < 0.05).

### Overall changes in prevalence and intensity of *S*. *haematobium* infection following treatments and reinfection

**Fig 3:**
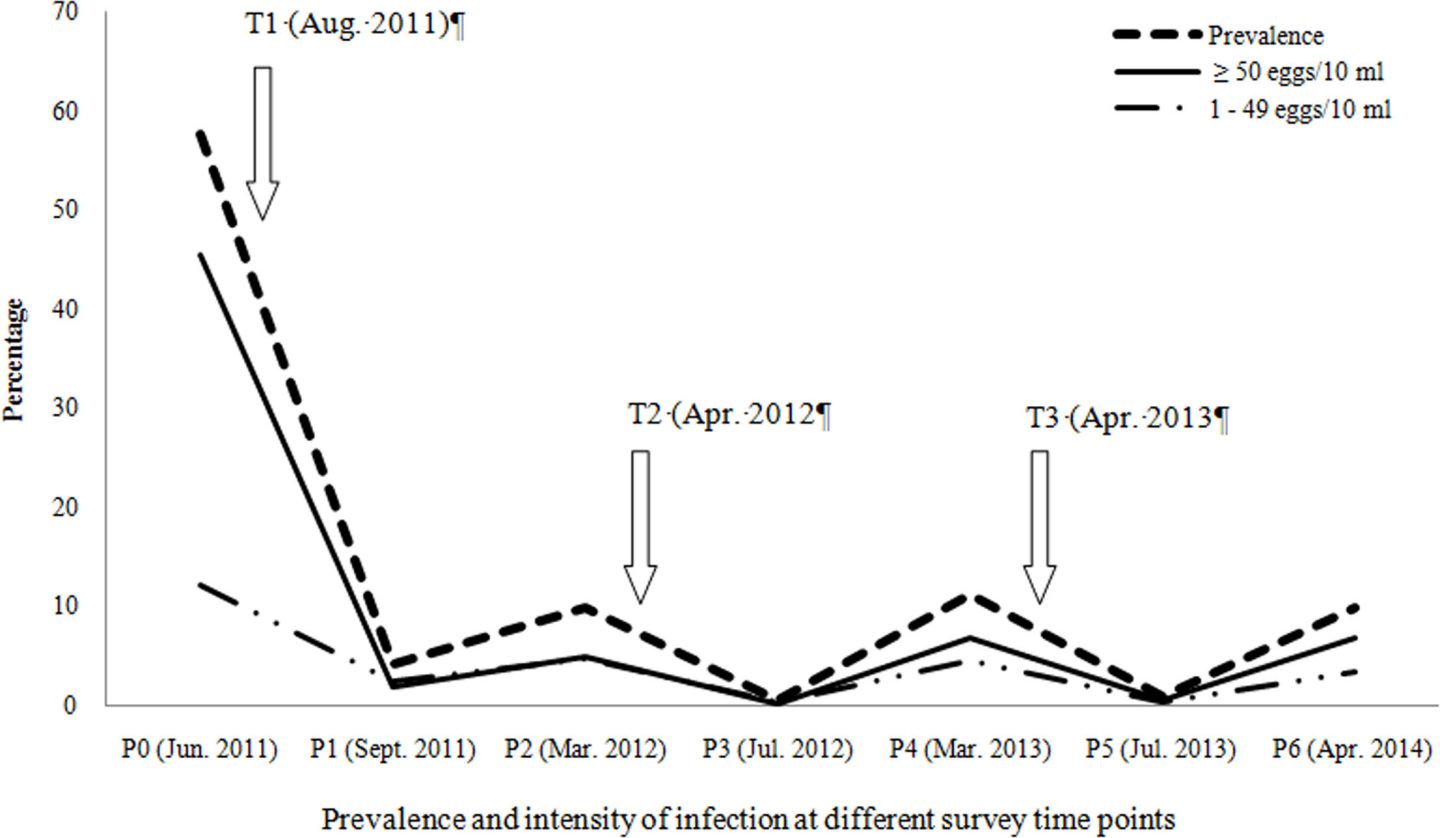
Graphical representation of variation in prevalence and intensity of *S*. *haematobium* infection in the study area after each treatment and reinfection from 2011 to 2014. T1, T2 and T3 indicate the three treatment of PZQ 40 mg/kg. S0 indicates the baseline survey time points. S1, S3 and S5 indicate the time points of the evaluation of efficacy of the treatments T1, T2 and T3 respectively. S2, S4 and S6 indicate the time points of the assessment of reinfection R1, R2 and R3 respectively.

After T1, the baseline (S0), prevalence (P0) was greatly reduced from 57.6% to 4.2% (P1) at S1 (*p*< 0.05). Overall heavy infection decreased from 45.4% at S0 to 1.8% at S1 (*p*< 0.05). During the follow-ups, the overall prevalence fluctuated following the treatment and the reinfection periods. During the S0 phase, the prevalence (P0) was high (57.6%) and it was reduced to 9.9% at S1, 11.2% at S2, and 9.9% at S3. Additionally, heavy intensity infections dropped from baseline (45.5%) to 4.9% at S1, 6.7% at S2 and 6.8% at S3 (Fig 3). It was also observed that after each transmission period, male SAC were significantly more frequently re-infected than the other groups (Table 3).

### Cercarial shedding from fresh water Bulinid snails from 2011 to 2013

From 2011 to 2013, 10,798 Bulinid snail vectors were collected from 13 ponds and a backwater frequented by the populations of the nine villages surveyed. All collected snails belonged to the species *B*. *senegalensis* (96.4%) or *B*. *umbilicatus* (3.6%). A total of 6,948 were tested for cercarial shedding with an overall infection rate of 34 (0.49%).

In 2011, none of the 530 snails tested shed schistosome cercariae. In 2012, the overall rate of infection was 0.8% in the 979 tested snails. However, only *B*. *umbilicatus* shed cercariae and all the infected snails were collected from a pond associated with V6. In 2013, the overall prevalence decreased compared to 2012 (*p* > 0.05) and 0.5% of the 5439 snails collected were positive. Positive *B*. *senegalensis* were found at V1, V4, V7 and V8, with prevalences of 0.7%, 1.1%, 2.6%, 0.05% and 0.6%, respectively. Positive *B*. *umbilicatus* snails were only found at V6, with an infection rate of 2.6% (Table 4).

**Table 4:** Global results of the malacological survey in water bodies from 2011 to 2013 in the nine villages selected in the Niakhar study area.

Villages													
Snail Survey	Infestation rates	V1	V2	V3	V4	V5	V6	V7	V8	V9	Total		
T1 (August 2011); transmission period June to November 2011													
September—December 2011	No. Snails tested	186	33	0	56	127	53	34	25	16	530		
	No. Snails infested (%)	0	0	0	0	0	0	0	0	0	0		
T2 (April 2012); December 2011 to July 2011 (water bodies are dry)													
July—December 2012	No. Snails tested	280	64	0	80	156	224	111	20	44	979		
	No. Snails infested (%)	0	0	0	0	0	8 (3.5)	0	0	0	8 (0.8)		
T3 (May 2013); December 2012 to July 2013 (water bodies are dry)													
July—December 2013	No. Snails tested	750	270	0	90	714	544	1920	819	330	5439		
	No. Snails infested (%)	5 (0.67)	0	0	1 (1.1)	0	14 (2.6)	1 (0.05)	5 (0.6)	0	26 (0.5)		
Total	No. Snails tested	1216	367	0	226	997	821	2065	864	390	6948		
	No. Snails infested (%)	5 (0.41%)	0	0	1 (0.44%)	0	22 (2.68%)	1 (0.048%)	5 (0.58%)	0	34 (0.49%)		

## Discussion

This study is the first large-scale investigation evaluating the efficacy of three successive annual treatments with a single dose of PZQ in a seasonal transmission focus of *S*. *haematobium* in Senegal. The study took place during three successive transmission cycles from 2011 to 2014. In 2011, before repeated treatments began, high prevalence and intensity of *S*. *haematobium* infection was recorded in all villages. However, only one urine sample per individual was examined at each time point due to limited resources and it is likely that prevalence levels could have been higher if duplicate urine samples were examined on different days to take into account day-to-day variation in egg excretion in urine [24]. However, our findings confirm the high endemicity of urogenital schistosomiasis in the Niakhar district [16] because no MDA has been carried out in this area before this study. This high prevalence of *S*. *haematobium* was exacerbated by the lack of health education and poor hygiene conditions in the study area. The epidemiology of urogenital schistosomiasis in Niakhar before PZQ MDA is characteristic of areas where the *S*. *haematobium* transmission is seasonal [11, 25, 26].

After only one round of treatment (T1), the overall prevalence and intensity of *S*. *haematobium* was significantly reduced. Heavy *S*. *haematobium* infections were largely eliminated, confirming the efficacy of a single 40 mg/kg dose of PZQ in this endemic setting [12]. A similar impact of one 40 mg/kg dose of PZQ was observed in populations of Diatar and Guia in the SRB [27], in Burkina Faso [28], and in Tanzania [29, 30]. The CR1 and the reduction of heavy intensity infections in the current study were more efficient than those observed in previous studies conducted in seasonal *S*. *haematobium* transmission foci [25]. Despite the fact that T1 was performed in during the period of transmission (August) due to the non-availability of PZQ in June, the CR was high for T1 (92.9%). The high efficacy of T2 and T3 was probably due to the low numbers of infected participants following the effectiveness of the 1st treatment (T1). A similar long-term impact of single PZQ treatment on *S*. *haematobium* infection was also reported two, three and six years after treatment in previous studies conducted in Tanzania [31], Niger [32] and in Kenya [33], respectively. We also observed the near elimination of high intensity infections after each treatment in the majority of the villages.

At the village level, the mapping of the hydro-geographical system showed differences in the type of water bodies used but also the proximity to the transmission sites to households, as indicated in Fig 1. A previous study of these water bodies also showed differences in the distribution and dynamics of the snail intermediate hosts *B*. *umbilicatus* and *B*. *senegalenis* from one year to another, both within and between villages [18]. Except for V6 where moderate CR1 was obtained, the efficacy of PZQ against *S*. *haematobium* in other villages was highly satisfactory. The lowest CR1 observed, in V6, may be due to a rapid reinfection due to the presence of *B*. *umbilicatus* in the pond used by the Ngangarlam population [18]. *B*. *senegalensis* was found in the fresh-water bodies associated with the other villages and were not found infected at the time of T1, supporting the low re-infection rates or presence of juvenile worms, which are not susceptible to PZQ, at the time of treatment. The results are consistent with other studies in permanent transmission areas with a high and low transmission period. The authors recommend treating in the low transmission period [34]. In the case of Niakhar district, the best time for MDA is in the non-transmission period between March and May.

After the first round of treatment, different patterns of reinfection were noted in the three villages using the backwater. The absence of re-infection in V3 may be due to the fact that the water is increasingly salty in the vicinity of this village and so is not a preferred snail habitat. In fact, in recent years, an increase in salinity was observed in the backwaters from the north to the south of the Niakhar area, which will help break transmission and could be a factor enabling elimination such as that seen in V3. The V2 inhabitants use the same sites in the backwater as V3 but also access a pond inside the village, which could be a source of reinfection. In contrast, in V1, situated in the north, the water from the backwater is still fresh allowing the development of snail intermediate hosts and maintenance of transmission. Differences were also noted after T1 in the other villages that only use freshwater ponds. Low reinfection rates were observed in V4, V5 V8 and V9 that have few ponds but also have access to tap water, thereby reducing human contact with infested water. By contrast, in V6 and V7, transmission appears to be more dynamic as the highest reinfection rates at R1 were recorded in these villages. This difference could be explained for V6 by the lack of safe drinking water and also the presence of *B*. *umbilicatus*. V7 had access to safe drinking water but high densities of *B*. *senegalensis* and close proximity of ponds [18]. In general, the levels of reinfection recorded in our villages after T1 were lower than those recorded after one treatment in *S*. *mansoni* or mixed *S*. *mansoni*/*S*. *haematobium* permanent foci in the ‘lac de Guier’ and in the SRB where prevalence after reinfection can rapidly return to pre-treatment levels [9, 10]. In this setting, tentative biological control of snails by native river prawns (*Macrobrachium vollenhoveni*) in combination with MDAs is being evaluated and as an approach to reduce the level of reinfection [35].

After the second treatment in 2012, the same general trends of reinfection were observed with a slight increase of R2 compared to R1, particularly in villages V6 and V7.

However, after the third treatment in 2013, R3 was lower than R1 and R2, suggesting that the result of the repeated treatments resulted in a considerable reduction in the number of viable eggs excreted into the environment and consequently a reduction in snail contamination at the transmission sites. The impact of repeated MDAs on snail infestation was strongly observed in V6, where a significant reduction in the rate of infested *B*. *umbilicatus* from 13.79% in 2012 to 4.98% in 2013 as previously reported [18].

The control of urogenital schistosomiasis with repeated annual PZQ treatment in this seasonal transmission setting has proven beneficial for the long-term goal of reducing morbidity and infection prevalence. Through the three years of study, although reinfection occurred in all the villages except V3, the rates were generally low. The ecology of the hydro-geographical system of the area of Niakhar probably plays a major role in the dynamics of *S*.*haematobium* reinfection.

Because 0.5% *Bulinus* snails were still shedding cercariae in five of the nine villages surveyed in 2013, transmission is ongoing. Therefore, the findings suggest that for long term control of urogenital schistosomiasis, the national program should integrate MDA with other control efforts such as health education and snail control to achieve the cessation of transmission in the Niakhar study area and other similar foci in Senegal.

### Conclusion

Repeated treatments have a considerable impact on the transmission of *S*. *haematobium* in Niakhar, due to the nature of the endemic foci, which have strictly seasonal transmission. Continuation of MDA in SCA, will allow this benefit to be maintained and to reduce the morbidity of *S*. *haematobium* in Niakhar area with a further move towards elimination. However, this study suggests that other strategies such as health education, improvement of access to clean water and snail control should be integrated into the strategy plans of the National program, to achieve the goal of urogenital schistosomiasis elimination from seasonal transmission foci in Senegal.

## Supporting Information

S1 Checklist STROBE checklist. (DOC)
